# LncRNA AFAP1-AS1 mediates therapy-dependent expression of CCL5, CXCL10 and MMP9 in multiple sclerosis

**DOI:** 10.1016/j.bbrep.2026.102595

**Published:** 2026-04-26

**Authors:** Lina Nasser Hegazy, Aya Aly Elkhodiry, Mohamed Hamed Rashad, Ramez Reda Moustafa, Hend Mohamed El Tayebi

**Affiliations:** a Department of Pharmacology and Toxicology, Faculty of Pharmacy and Biotechnology, German University in Cairo, Cairo, Egypt; b Department of Neurology, Faculty of Medicine, Al-Azhar University, Cairo, Egypt; c Department of Neurology, Faculty of Medicine, Ain Shams University, Cairo, Egypt

**Keywords:** Multiple sclerosis, M2 macrophages, LncRNAs, AFAP1-AS1, CXCL10, CCL5, MMP9

## Abstract

Multiple sclerosis (MS) is a chronic inflammatory autoimmune disease of the central nervous system. It is characterized by inflammation, areas of demyelination and axonal loss called plaques, recruitment of lymphocytes and monocytes, and bursts of focal blood-brain barrier leakage. Treatment strategies for MS focus on delaying disease progression and increasing patients’ quality of life. However, most therapies have inconsistent efficacies and are associated with various side effects. Recently, long non-coding RNAs have been found to play a major role in the pathogenesis and development of several diseases. Several long non-coding RNAs have been correlated with MS. We focus on the role of AFAP1-AS1 in regulating the function of M2 macrophages, one of the immune cells believed to attenuate MS. Assessing this long non-coding RNA will improve our understanding of the molecular mechanics of immune cells in MS. We observe the impact of AFAP1-AS1 silencing in M2 macrophages on essential effector and regulatory proteins like MMP9, CCL5 and CXCL10 in MS patients receiving different treatments (Fingolimod, Interferon beta-1a, Interferon beta-1b, Teriflunomide or Dimethyl fumarate). Our results reported an upstream regulatory effect of AFAP1-AS1 on MMP9, CCL5, and CXCL10 in differently treated patients. By measuring the levels of proteins upon silencing of AFAP1-AS1, it was confirmed that this lncRNA has varying effects on the expression of these proteins depending on the treatment the patient is undergoing. These data shed light on the potential role of manipulating the anti-inflammatory activity of M2 cells making it a possible therapeutic target for certain MS patients.

## Highlights

• AFAP1-AS1 is upregulated in M2 macrophages of differently treated MS patients.
• Silencing of AFAP1-AS1 alters MMP9, CCL5, and CXCL10 expression in M2 cells.
• Protein expression to AFAP1-AS1 silencing varies according to the type of MS treatment.
• Fingolimod and Interferon beta-1a groups showed MMP9 upregulation after silencing of AFAP1-AS1.
• AFAP1-AS1 may serve as a therapy-specific regulatory target in MS.

## 1 Introduction

Multiple sclerosis (MS) is a common neurological disorder typically presenting between ages 20 to 40 [1] with women affected 2-3 times more often than men [2]. It's characterized by inflammation, immune cell infiltration, and bursts of focal blood-brain barrier (BBB) leakage leading to demyelination, axonal destruction and loss of neurological function [3]. MS is classified into four main subtypes: Clinically isolated syndrome (CIS), Relapsing-Remitting MS (RRMS), Secondary-Progressive MS (SPMS) and Primary-Progressive MS (PPMS) [4].

Immune dysregulation in MS is multifactorial, involving genetic susceptibility, epigenetic, post genomic, environmental and immune factors [5]. MS pathogenesis begins with peripheral T and B cells activation, their migration across the BBB, subsequent CNS inflammation and tissue damage. Macrophages are key players in this process, exhibiting elevated levels in active demyelinating and early remyelinating lesions [6]. These cells undergo polarization into either pro-inflammatory M1 macrophages, present predominantly in early experimental autoimmune encephalomyelitis (EAE) or anti-inflammatory M2 macrophages, which promote tissue remodeling [[7], [8], [9]]. Despite evidence suggesting a protective role for M2 macrophages in MS, the regulatory mechanisms governing their immunosuppressive function remain unexplored.

Immune cells contribute to MS pathogenesis by expressing immunomodulatory proteins such as CCL5 and CXCL10. CCL5 also known as RANTES (regulated upon activation, normal T‐cell expressed and secreted), is a potent chemoattractant for monocytes, macrophages, microglia and T cells and is highly expressed by activated T cells, macrophages and microglia in active demyelinating lesions [10,11]. Elevated CCL5 levels in the cerebrospinal fluid (CSF) correlate with acute MS [12] particularly during relapses [13]. In EAE models, CCL5 facilitates macrophage entry and demyelination [14].

CXLC10, is a chemotactic agent for T helper type 1 (Th1) and natural killer (NK) cells to inflammation sites via CXC chemokine receptor 3 (CXCR3) binding [15]. It's expressed by monocytes, dendritic cells, astrocytes and endothelial cells [[16], [17], [18], [19], [20]] and is significantly elevated in CSF of patients with active MS [21]. Immunocytochemical studies established CXCL10 presence in postmortem CNS tissues of MS patients, predominantly in macrophages and reactive astrocytes within demyelinating lesions [22,23].

Immune cells also secrete matrix metalloproteinases (MMPs), a family of zinc containing and calcium requiring endopeptidases capable of degrading and remodeling the extracellular matrix (ECM) thereby affecting cell proliferation, differentiation, migration and death [24]. Among these, MMP9, is expressed by macrophages, osteoclasts and neutrophils and plays a crucial role in ECM degradation in physiological and pathological processes involving tissue remodeling [25]. MMP9 mRNA expression is increased in spinal cord of EAE mice and its inhibition confers partial resistance against EAE [26]. MMP9 levels were elevated in the serum and CSF of MS patients [27] indicating its involvement in disease activity.

Emerging evidence highlights the involvement of long non-coding RNAs (LncRNAs), a diverse group of non-coding RNA transcripts longer than 200 nucleotides [28], in immune regulation and MS pathogenesis [29]. Transcriptional regulatory networks can significantly influence the immune microenvironment by modulating immune cell infiltration, signaling pathways, and cellular metabolism. For example, the transcriptional regulator YY2 has been associated with alterations in immune cell infiltration and metabolic pathways, illustrating how gene-regulatory mechanisms shape immune responses in disease contexts [30]. Non-coding RNAs, including microRNAs (miRNAs), long non-coding RNAs (lncRNAs), and circular RNAs (circRNAs), play important roles in regulating gene expression at both transcriptional and post-transcriptional levels and have been associated with neurological disorders such as Alzheimer's disease [31]. Additionally, lncRNAs can form functional regulatory axes with microRNAs and effector proteins to modulate processes such as inflammation and autophagy, highlighting their broader roles in controlling cellular homeostasis and immune responses [32]. Beyond these roles, long non-coding RNAs can also act as competing endogenous RNAs (ceRNAs), interacting with microRNAs and downstream targets to regulate gene expression and key metabolic processes, further demonstrating their functional versatility in pathological regulation [33,34].

Actin filament-associated protein 1 antisense RNA 1 (AFAP1-AS1), a conserved lncRNA [35,36], regulates expression of the actin filament-associated protein 1 (AFAP1) at the translational level which links Proto-oncogene tyrosine-protein kinase Src (SRC) and Protein Kinase C (PKC) **—** crucial proteins in preserving BBB integrity [37]. Src kinases participate in several signaling pathways and help in signal propagation to the cytoskeleton, membrane, and nuclear targets. Activated Src kinase controls vascular endothelial growth factor (VEGFA) expression through the signal transducer and activator of transcription 3 (STAT3) pathway [38]. As VEGFA contributes to BBB dysfunction, Src likely promotes BBB leakage in MS, facilitating immune cell infiltration and subsequent demyelination [39,40].

AFAP1-AS1 has been linked to MS through bioinformatics analyses demonstrating its influence on small GTPases of the Rho/Rac pathway, critical for actin cytoskeleton signaling [41,42]. Dysregulation in their pathways may drive MS progression [43]. Consequently, AFAP1-AS1 might take part in MS progression by regulating AFAP1 expression or by affecting small GTPases. In fact, AFAP1-AS1 was found upregulated in peripheral blood of RRMS male MS patients compared to healthy controls [44].

While there is no cure for MS, current medications slow the disease progression and improve patients' quality of life, however, their efficacy and tolerability vary among individuals. Due to the complexity of MS and the individual variability in response to treatment, novel therapeutic approaches, such as genetic manipulation and ex-vivo approaches, are needed to improve treatment responses, and reduce adverse effects. This study examines the role of AFAP1-AS1 in M2 macrophages of MS patients receiving different treatment regimens due to its emerging role in MS pathogenesis. By analyzing the expression of key immunoregulatory proteins, known to play critical roles in MS development including MMP9, CCL5 and CXCL10, this work aims to investigate how AFAP1-AS1 regulates the function of M2 cells. Thus, our research represents a novel attempt to functionally analyze AFAP1-AS1 role in M2 cells of differently treated MS patients and highlight its potential as a therapeutic target for MS management.

## 2 Materials and methods

Detailed descriptions of all reagents, materials, and protocols are available in the Supplementary Data.

### 2.1 Sample collection

Blood samples were collected from 72 MS patients at the MS Unit, Nasser Institute for Research and Treatment following their written-informed consent and the ethical approval by the German University in Cairo Ethics Review Committee (Cairo, Egypt). The study protocol was approved by the Ethics Review Committee of the German University in Cairo (Approval Code: PTX-2020-06, Approval Date: 28/6/2020) and was conducted in accordance with the principles of the 1975 Declaration of Helsinki. Participants were aged between 20 and 60 years, diagnosed with MS, and undergoing treatment. Patients experiencing an active relapse during sampling were excluded. 10 age-matched healthy individuals served as controls.

### 2.2 Ficoll density gradient technique

10 ml blood was collected in EDTA tubes and 3 ml plasma was stored for further analysis. Peripheral blood mononuclear cells (PBMCs) were isolated within 4 h using Ficoll according to manufacturer's guidelines. Cells from the buffy layer underwent two washes in Rosewell Park Memorial Institute Medium-1640 (RPMI-1640), supplemented with l-glutamine, Phenol red, 5% FBS and 1% Penicillin/Streptomycin. Viable cells were counted and cryopreserved at −80 °C for up to 6 months. Viability was assessed upon thawing using 0.4% Trypan blue with >80% considered acceptable.

### 2.3 CD14^+^ monocytes isolation by negative depletion using magnetic nanobeads

PBMCs were thawed at 37 °C, transferred to 10 ml supplemented media and centrifuged. CD14+CD16^−^monocytes were isolated through negative depletion using the MojoSort™ Human CD14^+^ Monocytes Isolation Kit, MojoSort™ Buffer (5X) and MojoSort™ Magnet, following manufacturer's instructions. Isolated CD14^+^ monocytes were subsequently centrifuged and re-suspended in culture media.

### 2.4 Cell culture and differentiation

Freshly isolated CD14^+^ monocytes were resuspended at 1x10^6^ cells/ml in M2 differentiation medium consisting of supplemented media containing 100 ng/ml macrophage colony stimulating factor (M-CSF) and 10 ng/ml Interleukin 4 (IL-4) and from day 4, 10 ng/ml Interleukin 10 (IL-10). Cells were cultured in 48 well plates (10,000 cells/well) and incubated at 37 °C with 5% CO_2._ Media were refreshed every other day and by day 7, cells were harvested for transfection.

### 2.5 Flow cytometry

Confirmation of CD14^+^ monocytes isolation and M2 cells differentiation was performed via flow cytometry. Cells were dissociated and prepared as single-cell suspension (240,000 cells/tube), washed with 2 ml (PBS 1% FBS) and centrifuged. Cells were then incubated with 1.2 μg anti-CD163 FITC for 30 min at 4 °C followed by washing and acquisition. Flow cytometry analysis was conducted using CytoFLEX benchtop flow cytometer gating for the CD-163+ population. The acquired fluorescence data were analyzed using the CytExpert software to confirm purity. Regarding the isolation process and size scatter of the resultant populations, of note, 30% of the population consisted of residual monocytes and other T-cells that weren't completely depleted. Although negative depletion was used to isolate CD14^+^ monocytes, a residual population of non-monocyte cells remained. In addition, only a subset of differentiated cells expressed the CD163 marker, indicating partial heterogeneity within the macrophage population. Such heterogeneity is commonly reported in primary macrophage cultures derived from patient PBMCs and may arise from differences in monocyte isolation methods, polarization states, and donor-dependent variability [45,46]. While this may influence the magnitude of measured protein expression, the consistent trends observed across treatment groups support the regulatory role of AFAP1-AS1 in these cells. Importantly, the isolation and differentiation procedures were applied consistently across all treatment groups, supporting the comparability and validity of functional assays performed on these cells.

### 2.6 Transfection

Differentiated M2 macrophages harvested on day 7 were treated with 2 μl siRNA targeting lncRNA AFAP1-AS1 (Hs_MGC1098_3 FlexiTube siRNA; cat. no. SI04767511, Qiagen GmbH) diluted in 60 μl serum-free culture medium. HiPerfect Transfection Reagent (1 μl) was added to the diluted siRNA, mixed by vortexing, and incubated for 5–10 min at room temperature to allow formation of transfection complexes. The complex was then added dropwise onto the cells with gentle swirling. In addition, scrambled non-targeting siRNA was used as a negative control to account for potential non-specific effects of siRNA transfection (cat. no. 1027292, Qiagen GmbH). Transfections were conducted in triplicates and repeated three times per manufacturer's protocol. Cells treated with transfection reagent only were designated as mock cells, cells treated with scrambled non-targeting siRNA (negative controls) were designated as siNC and cells transfected with siRNA against AFAP1-AS1 were designated as siAFAP1-AS1 cells. After 6 h of incubation, 140 μl complete RPMI medium containing serum and antibiotics was added, and cells were incubated under normal conditions. Cells were lysed 48 h post-transfection for RNA extraction. The exact siRNA sequence is proprietary to the manufacturer and therefore not publicly disclosed. FlexiTube siRNAs are bioinformatically designed and experimentally validated by the manufacturer to minimize potential off-target effects.

### 2.7 RNA isolation

RNA extraction from cultured M2 macrophages was performed using RNeasy Minikit following the recommended extraction protocol. Total RNA was stored at −80 °C for later quantification of genes. RNA concentration and purity were calculated using Nanodrop, with acceptable A_260/280_ ratio between 1.9 and 2.2. For each sample, 30-50 ng RNA was utilized. RNA integrity was tested by 18s rRNA band detection using 1% agarose gel electrophoresis.

### 2.8 Reverse transcription of total mRNA into cDNA

Total extracted RNA was reverse-transcribed into single-stranded cDNA using the high-capacity cDNA reverse transcription kit following the manufacturer's instructions. Each 20 μl reaction consisted of equal volumes of RNA and reaction mix and were placed in a thermocycler with a heated lid using the recommended thermal profile. All cDNA samples were stored at −20 °C until RT-qPCR was performed.

### 2.9 Quantitative polymerase chain reaction (RT-qPCR) of AFAP1-AS1

The relative expression of AFAP1-AS1 normalized to GAPDH as a housekeeping gene, was quantified and amplified through TaqMan RT-qPCR for AFAP1-AS1 and GAPDH respectively using a StepOne™ Real-Time PCR instrument. AFAP1-AS1 probes were labeled with a FAM reporter dye while GAPDH probes with a VIC reporter dye. Each 20 μl reaction mix contained 4 μl nuclease-free water, 10 μl Premix Ex Taq™ (Probe qPCR), 1 μl TaqMan target gene expression assay, 1 μl GAPDH and 4 μl of the respective cDNA following the manufacturer's instructions. The RT-qPCR run was conducted in the standard mode, consisting of an initial 10-min stage (95 °C) for Taq-polymerase activation, followed by a second stage of 40 amplification cycles (15 s at 95 °C and 60 s at 60 °C). All PCR reactions, including controls, were carried out in triplicates and relative expression of genes was determined using the 2^−ΔΔCq^ method.

### 2.10 Protein quantification of MMP9, CCL5 and CXCL10 in serum samples using ELISA

Protein release of MMP9, CCL5 and CXCL10 in cell culture supernatants was measured using the human ELISA kits (MMP9; CCL5; CXCL10) following manufacturer's instructions. Test principle applied in this kit is Sandwich enzyme immunoassay utilizing microtiter plates pre-coated with antibodies specific to each target protein. Absorbance was analyzed for each microwell at 450 nm and results were calculated by constructing a standard curve plotting the mean OD and concentration for each standard.

### 2.11 Statistical analysis

Gene expression results were represented in relative quantitation (RQ). All experiments were performed in triplicates and data was presented as mean ± standard error of mean (SEM). Prior to statistical analysis, data distribution was assessed for normality using the Shapiro–Wilk test, and homogeneity of variance was evaluated using Levene's test. Since the data satisfied the assumptions of normality and equal variance, Student's t-test and One-way ANOVA were applied without data transformation. Given the small sample sizes per group (n = 3–4), the results of normality testing should be interpreted with caution. However, parametric tests were deemed appropriate based on the available data and consistent with similar studies in the field. Statistical analyses were performed using GraphPad Prism 7.04 software. Student's unpaired T-test was used for comparisons between two different studied groups while One Way Anova followed by Dunette's test of multiple comparison was employed for comparisons among multiple different studied groups. A significance level of P < 0.05 was indicative of a statistically significant difference.

## 3 Results

### 3.1 Demographics of clinical patients

Patients were enlisted from Oct 2020 to March 2022. The mean age of patients was 32.97 years with an age range of 20-54 years. Samples were subdivided according to their treatments. 10 age matched healthy controls were employed and same experiments were done on their samples. Clinical parameters of patients and controls are presented in Table 1. Detailed clinical features of all patients and controls are mentioned in Supplementary Tables 1 and 2

**Table 1:** Characteristics of patients and healthy controls.

A, Patients (n = 72)	Percentage (%)
**Sex**	
Female (45/72)	62.5%
Male (27/72)	37.5%

**Age**	
< 50 years (70/72)	97.2%
>50 years (2/72)	2.7%

**Type**	
RRMS (70/72)	97.2%
SPMS (2/72)	2.7%
PPMS (0/72)	0%

**Treatment**	
Fingolimod (26/72)	36%
Interferon beta-1a (24/72)	33.3%
Interferon beta-1b (9/72)	12.5%
Dimethyl fumarate (8/72)	11.1%
Teriflunomide (5/72)	6.9%

**EDSS**	
1.0 – 1.5 (15/72)	20.8%
2.0 – 2.5 (20/72)	27.7%
3.0 – 3.5 (20/72)	27.7%
4.0 – 4.5 (12/72)	16.6%
5.0 – 5.5 (3/72)	4.1%
>5.5 (2/72)	2.7%

**MRI load**	
Low (17/72)	23.6%
Moderate (7/72)	9.7%
High (48/72)	66.7%

**B, Controls (n=10)**	**Percentage *(%)***
**Sex**	
Female (7/10)	70%
Male (3/10)	30%

**Age**	
< 50 years (10/10)	100%
>50 years (0/10)	0%

#### 3.1.1 Efficiency of CD14^+^ monocytes differentiation into M2 cells

Differentiation efficiency of CD14^+^ monocytes into M2 macrophages was confirmed by observing morphological change and flow cytometry.

#### 3.1.2 Flow cytometry

**Fig. 1:**
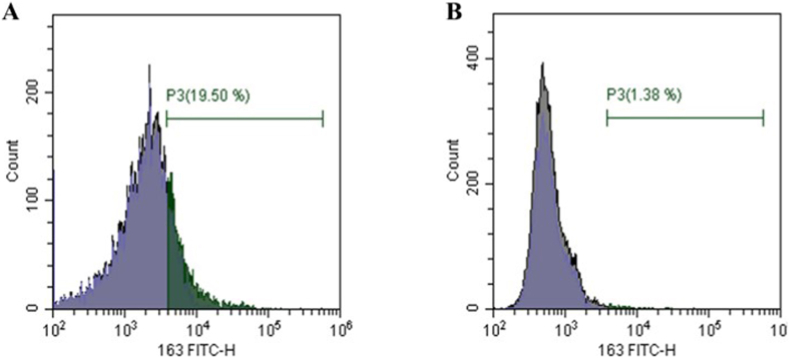
**Flow cytometry using anti-CD163**. Differentiation efficiency was analyzed using flow cytometry through the measuring of CD163 which a self-surface marker of M2 cells is. (A) Histogram shows 19.5% positive stained cells against anti-CD163 after culturing the CD14^+^ Monocytes for 7 days thus differentiation into M2 macrophages. (B) Histogram showing 1.38% of the freshly isolated CD14^+^ Monocytes (undifferentiated) were stained against anti-CD163. This baseline percentage reflects pre-differentiation levels and was not used in functional analyses.

Flow cytometry technique was conducted using anti-CD163 FITC, a self-surface marker for M2 cells. After 7 days of culturing CD14^+^ monocytes in the differentiation media (Culture media supplemented with IL10, IL4 and MCSF), 19.5% of cells were CD163^+^, compared to 1.38% representing the uncultured freshly isolated CD14^+^ monocytes, thus confirming successful differentiation as shown in Fig. 1. The 1.38% value was used solely to assess baseline expression in undifferentiated monocytes and does not reflect the M2 population used in downstream experiments.

#### 3.1.3 Morphological change

**Fig. 2:**
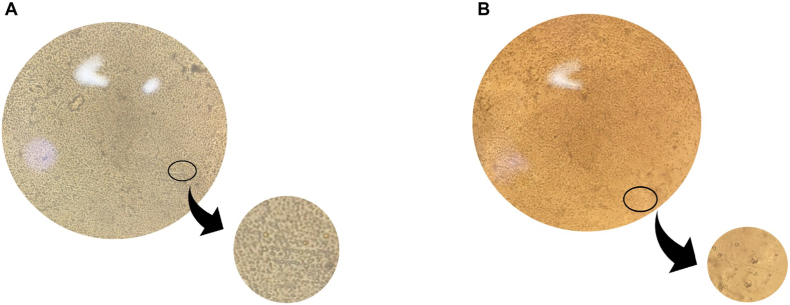
**Morphology of freshly isolated CD14^+^ Monocytes and M2 cells of MS under microscope**. (A) CD14^+^ Monocyte's morphology was examined under the microscope. Examination showed cells with spherical and smooth surfaces. Cells are considered small in size. (B) M2 cells morphology was examined under the microscope. Examination showed cells with edgy and rough surface and a relatively larger size than Monocytes.

Freshly isolated CD14^+^ Monocytes appeared as small, spherical shaped cells with a smooth surface under the microscope (Fig. 2A). However, after 7 days of culture in media supplemented with IL10, IL4 and MCSF, cells exhibited a larger size with non-smooth edges (Fig. 2B), indicating morphological differentiation.

### 3.2 Expression profile of AFAP1-AS1 in M2 cells of different MS treatment groups

**Fig. 3:**
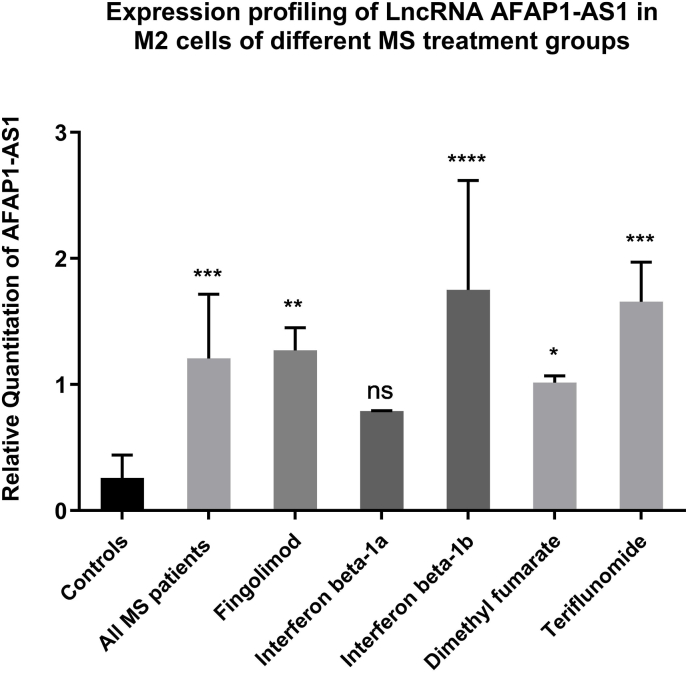
**Expression profiling of lncRNA AFAP1-AS1 in M2 cells of different treatment groups**. q-PCR was performed for the quantitative analysis and expression profiling of AFAP1-AS1 levels in M2 cells of different MS patients compared to healthy donors. Results show significant upregulation of AFAP1-AS1 in Fingolimod (n = 3), Interferon beta-1b (n = 3), Dimethyl fumarate (n = 4) and Teriflunomide (n = 4) treatment groups except with Interferon beta-1a (n = 3), compared to healthy donors (n = 10). ****p < 0.0001, ***p < 0.001, **p < 0.01, and *p < 0.05. n = number of patients.

Notably, this is the first study assessing AFAP1-AS1 expression in M2 cells of differently treated MS patients. The expression of AFAP1-AS1 was found to be upregulated in the Fingolimod, Interferon beta-1b, Dimethyl fumarate and Teriflunomide treatment groups (P = 0.0022, <0.0001, 0.0252, 0.0005 respectively) compared to healthy donors, with no significant change in the Interferon beta-1a group as shown in Fig. 3.

### 3.3 Screening of MMP9, CCL5 and CXCL10 protein levels in serum of MS patients

**Fig. 4:**
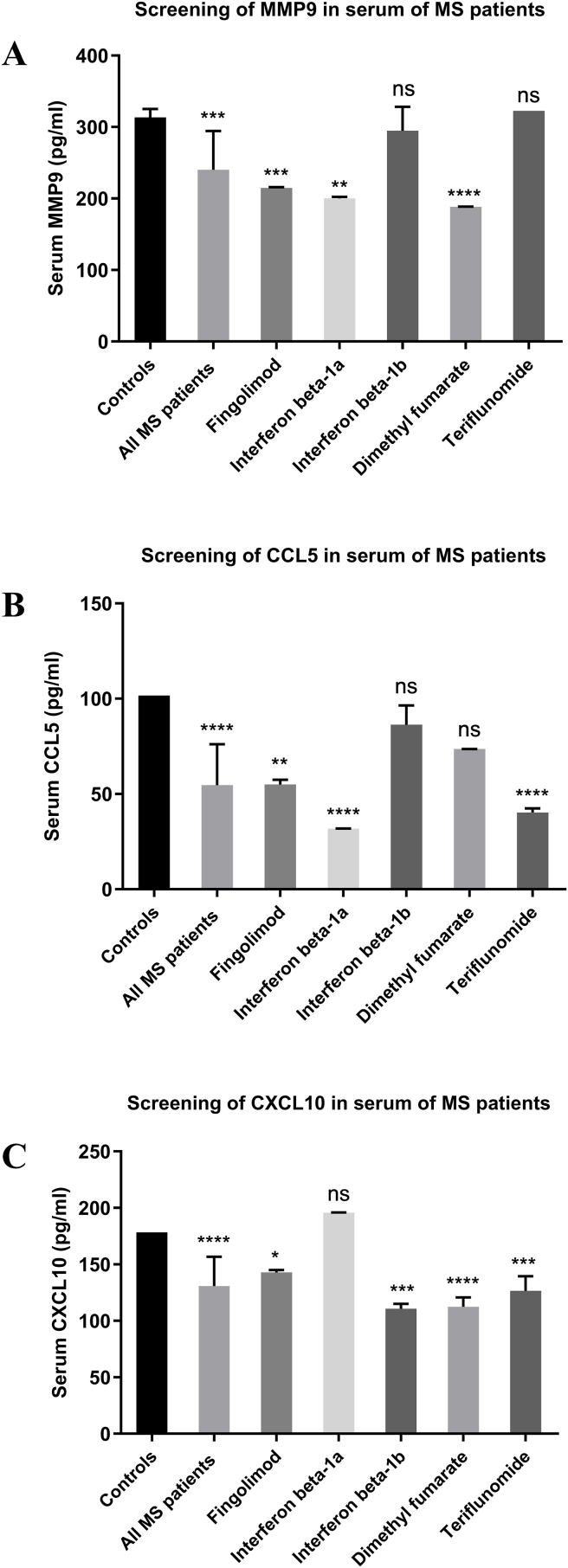
**Screening of MMP9, CCL5, CXCL10 in serum of MS patients.** ELISA method was used to measure the levels of MMP9, CCL5 and CXCL10 in serum of differently treated MS patients. Results showed (A) A significant downregulation of MMP9 protein release in serum of MS patients treated with Fingolimod (n = 3), Interferon beta-1a (n = 3) and Dimethyl fumarate (n = 4) against healthy donors (controls). While it showed no significant change in patients treated with Interferon beta 1b (n = 3) or Teriflunomide (n = 4) compared to the controls. (B) A significant downregulation of CCL5 protein release in serum of MS patients treated with Fingolimod (n = 3), Interferon beta-1a (n = 3) and Teriflunomide (n = 4) compared to healthy donors (controls) was observed. On the contrary, patients treated with Interferon beta-1b (n = 3), or Dimethyl fumarate (n = 4) showed no significant difference in CCL5 levels when compared to the controls. (C) A significant downregulation of CXCL10 protein release in serum of patients treated with Fingolimod (n = 3), Interferon beta-1b (n = 3), Dimethyl fumarate (n = 4) and Teriflunomide (n = 4) compared to healthy donors (controls) and no significant change in patients treated with Interferon beta-1a (n = 3) when compared to the controls. ****p < 0.0001, ***p < 0.001, **p < 0.01, and *p < 0.05. These results reflect systemic serum levels and are not derived from M2 macrophage cultures. n = number of patients.

Due to the overexpression of AFAP1-AS1 in M2 cells of MS patients, the impact of its knockdown on the expression of MMP9, CCL5 and CXCL10 was assessed. However, before investigating protein levels following AFAP1-AS1 silencing, their expression in serum of different MS patients were measured against controls. MMP9 protein expression was significantly downregulated in the serum of Fingolimod, Interferon beta-1a and Dimethyl fumarate groups (P = 0.0002, 0.0038, <0.0001 respectively) compared to controls, with no significant change in the Interferon beta-1b or Teriflunomide groups (Fig. 4A). CCL5 protein release was significantly reduced in the serum of patients treated with Fingolimod, Interferon beta-1a and Teriflunomide (P = 0.0011, <0.0001, <0.0001 respectively) in comparison to controls. However, there was no significant change in its levels in the Interferon beta-1b and Dimethyl fumarate groups (Fig. 4B). CXCL10 protein expression was significantly downregulated in patients treated with Fingolimod, Interferon beta-1b, Dimethyl fumarate and Teriflunomide (P = 0.0332, 0.0001, <0.0001, 0.0007 respectively) compared to controls, with no significant change in the Interferon beta-1a treated patients (Fig. 4C). It is important to note that these serum measurements represent systemic protein levels and were not attributed to M2 macrophages, which were used in subsequent in vitro experiments.

### 3.4 Transfection efficiency for AFAP1-AS1 silencing

**Fig. 5:**
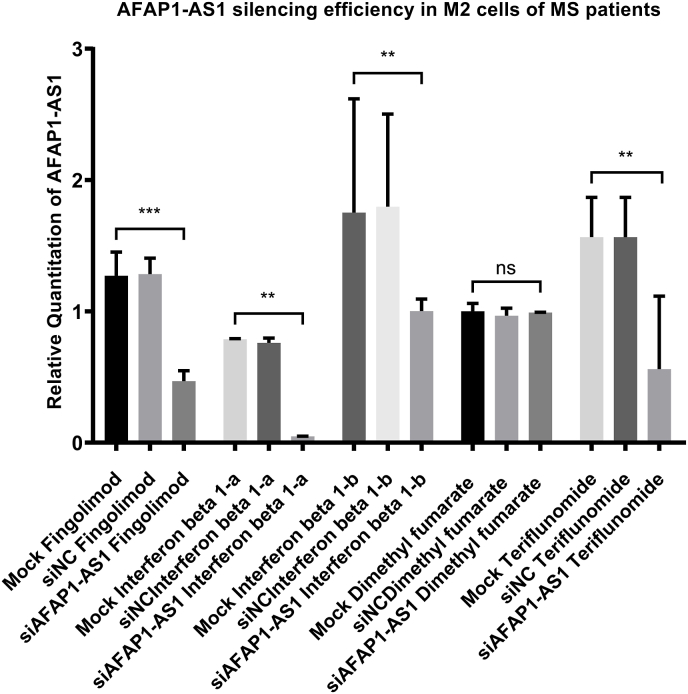
**AFAP1-AS1 silencing efficiency in M2 cells of MS patients**. q-PCR was performed for the quantitative analysis of AFAP1-AS1 expression in M2 macrophages from the 5 treatment groups following transfection. Cells were treated with siRNA targeting AFAP1-AS1 (siAFAP1-AS1), scrambled non-targeting siRNA as a negative control (siNC), or transfection reagent only (mock). Transfection with silencers against AFAP1-AS1 resulted in a significant decrease in expression in Fingolimod (n = 3), Interferon beta-1a (n = 3), Interferon beta-1b (n = 3), and Teriflunomide (n = 4) treatment groups, whereas no significant change was observed in the Dimethyl fumarate group (n = 4), compared with the corresponding mock controls. No significant changes were observed between siNC-treated cells relative to mock controls, confirming specificity of AFAP1-AS1 silencing. ****p < 0.0001, ***p < 0.001, **p < 0.01, and *p < 0.05. n = number of patients.

Transfection efficiency was first assessed 48 h post-transfection using RT-qPCR. Transfection of M2 cells with silencers against AFAP1-AS1 resulted in significant downregulation, thus silencing of AFAP1-AS1 expression in the Fingolimod, Interferon beta-1a, Interferon beta-1b and Teriflunomide groups compared with their corresponding mock controls (P = 0.0003, 0.0012, 0.0039, 0.0068 respectively), whereas no significant change in AFAP1-AS1 levels was observed in the Dimethyl fumarate group compared with mock controls, as shown in Fig. 5. Importantly, cells transfected with scrambled non-targeting siRNA (siNC) did not show significant differences in AFAP1-AS1 expression compared with the corresponding mock controls, indicating that the negative control itself did not affect AFAP1-AS1 expression and supporting the specificity of AFAP1-AS1 silencing.

### 3.5 Impact of AFAP1-AS1 silencing on MMP9, CCL5 and CXCL10 protein release from M2 cells of MS patients

**Fig. 6:**
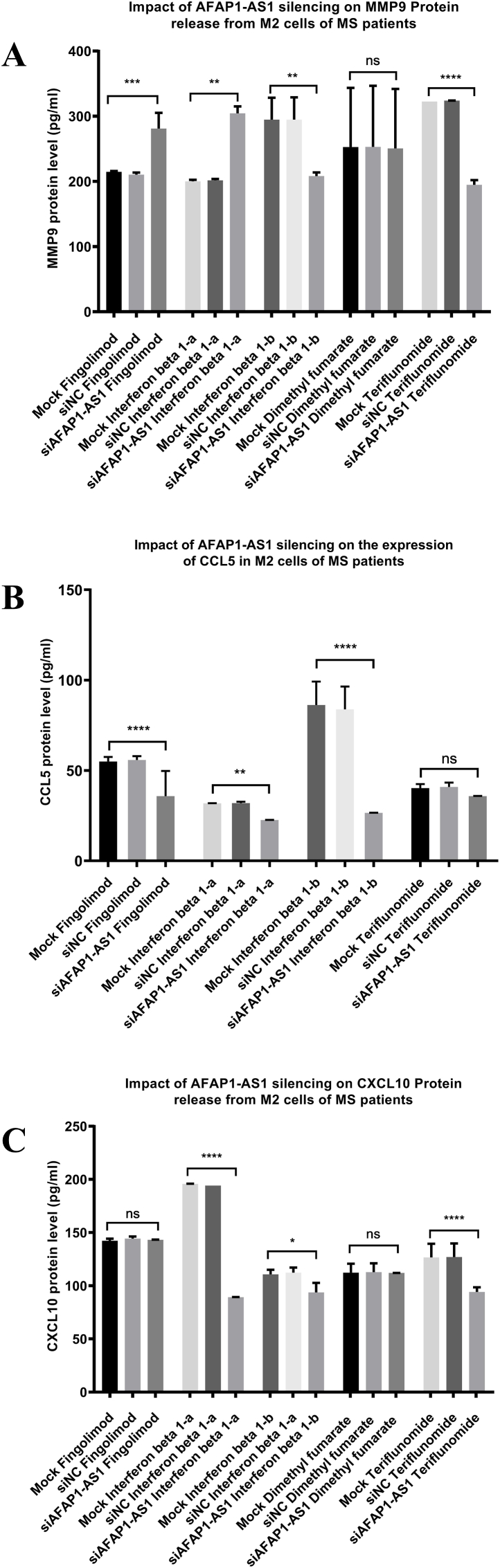
**Impact of AFAP1-AS1 silencing on MMP9, CCL5 and CXCL10 protein release from M2 cells of MS patients**. ELISA technique was used to measure the levels of MMP9, CCL5 and CXCL10 in the cell culture supernatants of differently treated MS patients. Scrambled non-targeting siRNA (siNC) showed no significant differences compared with the corresponding mock controls across the assessed markers, supporting the specificity of AFAP1-AS1-targeting siRNA and indicating the absence of detectable non-specific transfection-related effects. Therefore, the statistical comparisons presented in this figure are between mock and AFAP1-AS1-silenced groups only. Results showed that (A) AFAP1-AS1 silencing resulted in statistically significant downregulation of MMP9 protein release from M2 cells of MS patients treated with Interferon beta-1b (n = 3) and Teriflunomide (n = 4) compared with the corresponding mock controls. While it caused a significant upregulation of MMP9 levels in patients treated with Fingolimod (n = 3) or Interferon beta-1a (n = 3) in comparison to the corresponding mock controls. Patients treated with Dimethyl fumarate (n = 4) showed no significant change in MMP9 expression following AFAP1-AS1 silencing in M2 cells compared to mock controls. (B) AFAP1-AS1 silencing resulted in statistically significant downregulation of CCL5 protein release from M2 cells of MS patients treated with Fingolimod (n = 3), Interferon beta-1a (n = 3) and Interferon beta-1b (n = 3) compared with the corresponding mock controls. However, upon silencing of AFAP1-AS1 in M2 cells of MS patients treated with Teriflunomide (n = 4), no significant change in CCL5 levels was observed in comparison to the corresponding mock controls. (C) AFAP1-AS1 silencing resulted in statistically significant downregulation of CXCL10 protein release from M2 cells of MS patients treated with Interferon beta-1a (n = 3), Interferon beta-1b (n = 3) and Teriflunomide (n = 4) compared with the corresponding mock controls However, upon silencing of AFAP1-AS1 in M2 cells of MS patients treated with Fingolimod (n = 3) or Dimethyl fumarate (n = 4), no significant change in CXCL10 levels was observed in comparison to the corresponding mock controls. ****p < 0.0001, ***p < 0.001, **p < 0.01, and *p < 0.05. n = number of patients.

Scrambled non-targeting siRNA (siNC) showed no significant differences compared with the corresponding mock controls across the assessed protein markers, supporting the specificity of the AFAP1-AS1-targeting siRNA and indicating the absence of detectable non-specific transfection-related effects. Therefore, all comparisons reported in this section were made between mock and AFAP1-AS1-silenced groups only. Transfection of M2 cells with silencers against AFAP1-AS1, led to a significant decrease in MMP9 expression in the culture supernatants of patients treated with Interferon beta-1b and Teriflunomide (P = 0.0041, <0.0001 respectively). Interestingly, MMP9 levels were upregulated in M2 cells of Fingolimod and Interferon beta-1a patients (P = 0.0009, 0.0022 respectively). However, no significant change was observed in MMP9 expression in the Dimethyl fumarate group (Fig. 6A). CCL5 levels were also measured following AFAP1-AS1 silencing, and its expression was significantly reduced in the supernatants of the Fingolimod, Interferon beta-1a and Interferon beta-1b groups (P= <0.0001, 0.0030, <0.0001 respectively) while no significant change was observed in the Teriflunomide group compared to the corresponding mock control (Fig. 6B). Because AFAP1-AS1 silencing was not achieved in the Dimethyl fumarate group, CCL5 levels were not further assessed in this group. CXCL10 expression was significantly reduced upon silencing of AFAP1-AS1 in the supernatants of the Interferon beta-1a, Interferon beta-1b and Teriflunomide groups (P= <0.0001, 0.0288, <0.0001 respectively) however, it showed no significant change in either the Fingolimod or Dimethyl fumarate groups (Fig. 6C).

## 4 Discussion

MS is a chronic autoimmune disease of the CNS causing myelin loss and impaired signal transmission [47]. Current therapies suppress autoreactive immune cells, but show inconsistent efficacies and adverse effects [48], highlighting the need for more targeted therapy strategies. M2 macrophages, known for their anti-inflammatory and tissue-repair roles [49], release IL-10 and Transforming growth factor β (TGF-β) [50], suppress T cells and microglial activation while promoting regulatory T cell differentiation [51], suggesting a therapeutic potential in MS. Non-coding RNAs like AFAP1-AS1 are also implicated in MS due to the epigenetic changes observed during disease course [52]. AFAP1-AS1 regulates AFAP1, which maintains BBB integrity [37] and influences Rho/Rac signaling pathway, important in immune regulation, making it an intriguing target in MS [41,43].

This study investigates AFAP1-AS1 role in regulating pro-inflammatory proteins (MMP9, CCL5, CXCL10) in M2 cells of differently treated MS patients, given their role in MS pathogenesis [53]. To our knowledge, this is the first study assessing AFAP1-AS1 expression in M2 cells of MS patients. Its upregulation in MS and involvement in pathways affecting BBB integrity and cytoskeletal rearrangements suggest therapeutic potential.

AFAP1-AS1 was overexpressed in M2 cells in Fingolimod, Interferon beta-1b, Dimethyl fumarate, and Teriflunomide groups (Fig. 1), aligning with earlier findings [44]. Subsequently, serum protein levels were measured across treatment groups and controls.

MMP9 was downregulated in Fingolimod, Interferon beta-1a, and Dimethyl fumarate groups compared to controls. Fingolimod reduces BBB permeability hence limit inflammatory cells infiltration and subsequent MMP9 release [54]. This finding aligns with previous studies reporting downregulation in MMP9 gene expression in Fingolimod-treated EAE models [55]. Multiple studies showed that Interferon beta-1a lowers serum MMP9 levels [56,57], consistent with this study. Dimethyl fumarate has demonstrated a protective effect on the BBB by decreasing MMP activity through activation of nuclear factor erythroid 2-related factor (NRF2) pathway [58]. Although previous studies reported decreased MMP9 levels with Interferon beta-1b in RRMS [59] and PPMS [60], no significant change was observed here, likely due to differences in treatment duration, sample size, or disease pathology.

CCL5 expression was significantly downregulated in Fingolimod, Interferon beta-1a, and Teriflunomide, groups. Although one study reported increased CCL5 expression in Fingolimod-treated RRMS patients [61], other EAE models studies support its downregulation, possibly due to Fingolimod's effect on sphingosine-1-phosphate (S1P) receptors on lymphocytes, retaining them in lymph nodes. CCL5 downregulation with Interferon beta-1a group coincides with previous studies showing suppressed CCL5 mRNA synthesis [62,63]. Direct studies quantifying CCL5 levels following Teriflunomide treatment are lacking; however, its suppression of tumor necrosis factor α (TNF-α) and IFN-γ [[64], [65], [66], [67]] known CCL5 inducers [55,[68], [69], [70]] may explain the observed reduction.

CXCL10 expression was downregulated in all treatment groups, except Interferon beta-1a. Fingolimod's results corroborates studies showing suppression of TNF-induced CXCL10 [71]. Interferon beta-1b may reduce CXCL10 by shifting from a Th1 to Th2 profile [72] although some studies show increased CXCL10 with this treatment [73]. Previous studies show decreased CXCL10 mRNA and protein levels in human PBMCs following Dimethyl fumarate [74], matching our findings. Teriflunomide's reduction of CXCL10 may result from its interference with the nuclear factor kappa-light chain enhancer of B cells (NF-κB) pathway, a key CXCL10 regulatory pathway [75]. This is supported by reduced CXCL10 secretion in microglia following Teriflunomide treatment [76]. The insignificant change of CXCL10 with Interferon beta-1a conflicts with studies showing increased CXCL10 plasma [72,77] or mRNA levels [63,78,79] possibly due to differences in treatment duration, disease stage, or sample size. The next part of this study is to measure MMP9, CCL5, and CXCL10 levels after AFAP1-AS1 silencing in M2 cells of MS patients. The therapy-dependent effects of AFAP1-AS1 silencing can be understood through the distinct molecular contexts created by each disease modifying treatment, as detailed below for each protein and treatment group.

The effect of AFAP1-AS1 knockdown on MMP9 was measured via ELISA. AFAP1-AS1 silencing reduced MMP9 in Interferon beta-1b and Teriflunomide groups. A study by Hollborn et al., reported a positive feedback regulation between MMP9 and VEGF in retinal pigment epithelial cells, where VEGF enhances MMP9 expression and secretion [80]. Hence, AFAP1-AS1 silencing likely disrupts AFAP1-Src interaction, downregulating VEGF expression thus decreasing MMP9.

Contrarily, MMP9 increased in Fingolimod and Interferon beta-1a groups upon AFAP1-AS1 silencing, possibly due to a compensatory positive feedback loop. Fingolimod downregulates NF-κB [81] and IL-17 signaling [82] both linked to MMP9 inhibition [83] therefore, the additional disruption of AFAP1-Src signaling following AFAP1-AS1 silencing may further alter MMP9 regulatory pathways, potentially triggering a compensatory feedback response leading to increased MMP9 expression. No change was observed in Dimethyl fumarate group, possibly because its primary mechanism via nuclear factor erythroid 2–related factor 2 (NRF2) pathway activation doesn't affect MMP9 regulation [84].

**Fig. 7:**
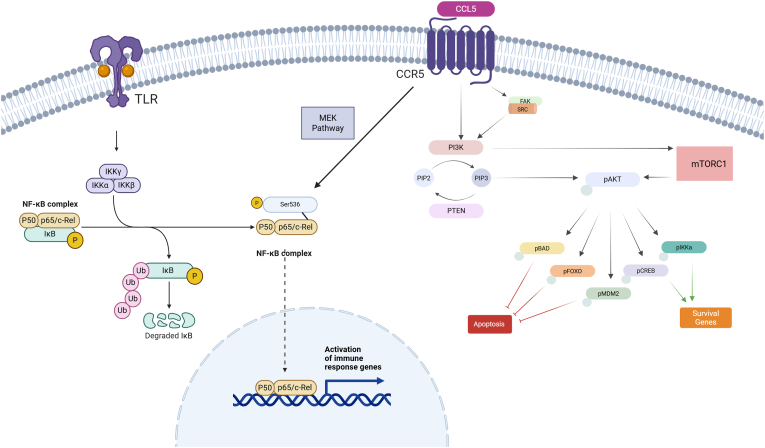
**CCL5/CCR5 axis in PI3K/AKT pathway and NF-κB pathway**. When CCL5 binds to its receptor CCR5, it mainly triggers the PI3K/Akt pathway, leading to the phosphorylation of PIP2 to PIP3, which is a secondary messenger that activates the Akt kinase. The Akt kinase then phosphorylates several downstream effectors. However, research has also demonstrated that a Focal Adhesion kinase (FAK) binding-Src kinase complex can be activated as a secondary intracellular target, resulting in further PI3K activation. Additionally, this binding phosphorylates p65 at serine 536 which triggers the phosphorylation cascade of IKK (inhibitor of nuclear factor-κB kinase) factor- and IκB (inhibitor of nuclear factor-κB) subunits, ultimately activating NF-κB. Following the degradation of phosphorylated IκB by ubiquitination, NF-κB enters the nucleus (created by Biorender.com).

CCL5 protein levels were significantly reduced upon silencing in Fingolimod, Interferon beta-1a and Interferon beta-1b. CCL5 binding to CCR5 activates the phosphatidylinositol 3-kinase/protein kinase B (PI3K/Akt) pathway and binding of Focal adhesion kinase (FAK) to Src kinase, resulting in further PI3K/Akt activation as shown in Fig. 7. Since AFAP1-AS1 regulates AFAP1, a key Src-binding partner, its silencing likely disrupts AFAP1–Src interactions, leading to reduced PI3K/Akt signaling and consequently decreased CCL5 expression [37,85]. This effect may be reinforced by the mechanisms of the studied therapies. Fingolimod modulates sphingosine-1-phosphate (S1P) receptors, limiting lymphocyte trafficking [86] and suppressing inflammatory signaling pathways [87]. Similarly, Interferon beta-1a and Interferon beta-1b reduce pro-inflammatory cytokine signaling and chemokine expression [88,89]. Thus, AFAP1-AS1 knockdown may act synergistically with these treatments to further suppress CCL5 production. AFAP1-AS1 also influences Rho GTPases, crucial for actin cytoskeleton remodeling [90,91]. Actin cytoskeleton is essential for antigen receptor activation and T cell polarization towards APCs and any disruption during this contact impairs T-cell signaling and reduces cytokine secretion [92]. Src-mediated phosphorylation of Rho GTPases [93] is impaired by AFAP1-AS1 silencing potentially disrupting antigen presentation and reducing CCL5 secretion.

CXCL10 levels decreased following AFAP1-AS1 silencing in Interferon beta-1a, Interferon beta-1b, and Teriflunomide groups. CXCL10 induction is driven by TNF-α-induced NF-κB transcriptional activation [94], with Rho proteins (RhoA and Rac1) acting downstream of TNF-α [95,96]. AFAP1-AS1 silencing likely inhibits this pathway by disrupting Rho-mediated signaling downstream of TNF-α, thereby reducing CXCL10 levels. This effect may interact with the immunomodulatory mechanisms of Interferon beta-1a, Interferon beta-1b and teriflunomide treatments, which also influence inflammatory signaling pathways [75,88,89]. Unexpectedly, Dimethyl fumarate-treated patients showed no reduction in CXCL10, despite its known NF-κB inhibitory effect and AFAP1-AS1 silencing was expected to enhance this inhibition suggesting possible involvement of alternative regulatory pathways maintaining CXCL10 expression [[97], [98], [99], [100], [101]].

Future recommendations should involve measuring mRNA levels of the 3 proteins before silencing of AFAP1-AS1 to account for post-transcriptional changes. While our current findings suggest a regulatory role for AFAP1-AS1 in modulating these markers in M2 macrophages, further mechanistic validation is warranted. Specifically, rescue experiments, pathway enrichment analyses, and chromatin interaction assays would provide deeper insight into the molecular mechanisms and clarify the direct signaling pathways involved. Moreover, larger longitudinal studies are recommended to validate these proteins as potential prognostic indicators or treatment response biomarkers. Additionally, evaluating gender-based differences in AFAP-AS1 expression and comparing these markers in M1 and M2 cells would provide further insight.

This study demonstrates that AFAP1-AS1 regulates MMP9, CCL5, and CXCL10 expression in M2 macrophages of MS patients in a therapy-dependent manner as shown in Table 2. This underscores the potential of AFAP1-AS1 as a regulatory mediator whose functional impact varies according to the disease-modifying therapy administered. These findings provide new insights into lncRNA-mediated immune regulation in MS, emphasizing AFAP1-AS1 as a potential therapeutic target.

**Table 2:** Summary of protein expression levels after silencing of AFAP1-AS1 in the M2 cells of different MS patients.

Treatment	Interferon beta-1b	Interferon beta-1a	Teriflunomide	Fingolimod	Dimethyl fumarate
Protein					
**MMP9**	Downregulation	Upregulation	Downregulation	Upregulation	No change
**CCL5**	Downregulation	Downregulation	No change	No change	No change
**CXCL10**	Downregulation	Downregulation	Downregulation	Downregulation	Downregulation

## Ethics approval and consent to participate

The studies involving human participants were reviewed and approved by German University in Cairo and Ethical Committee. The patients/participants provided their written informed consent to participate in this study.

## Availability of data and materials

All datasets generated for this study are included in the article/Supplementary Material.

## Funding

This research did not receive any specific grant from funding agencies in the public, commercial, or not-for-profit sectors.

## CRediT authorship contribution statement

**Lina Nasser Hegazy:** Conceptualization, Data curation, Formal analysis, Investigation, Methodology, Visualization, Writing – original draft. **Aya Aly Elkhodiry:** Methodology, Writing – review & editing. **Mohamed Hamed Rashad:** Resources. **Ramez Reda Moustafa:** Resources. **Hend Mohamed El Tayebi:** Conceptualization, Formal analysis, Investigation, Project administration, Supervision, Writing – review & editing.

## Declaration of competing interest

I have nothing to declare.

## Data Availability

All datasets generated for this study are included in the article/Supplementary Material.
