# Supplementary material for: LncRNA AFAP1-AS1 mediates therapy-dependent expression of CCL5, CXCL10 and MMP9 in multiple sclerosis
Source: Biochem Biophys Rep. 2026 Apr 26;46:102595. doi: 10.1016/j.bbrep.2026.102595 (PMC13137071; doi:10.1016/j.bbrep.2026.102595)
Supplement: Multimedia component 1 [file mmc1.docx]

**Supplementary Data**

**Supplementary Table 1: Clinical features of each MS patient.**

Clinical data of each patient including Sex, Age, Type of MS, EDSS, Therapy, Time from last relapse, Disease duration, MRI load, Disease activity, and Response to therapy.

| **Serial #** | **Ptn #** | **Gender** | **Age** | **MS Type** | **EDSS** | **Therapy** | **Time since last relapse** | **Disease Duration** | **MRI Load - High/Medium/Low(H/M/L)** | **Active or Inactive Disease (A/I)** | **Responsive or not responsive to treatment (R/N)** |
| --- | --- | --- | --- | --- | --- | --- | --- | --- | --- | --- | --- |
| 1 | 4 | Female | 36 | RRMS | 4.5 | Interferon beta-1a (Avonex & Rebif) | 6months | 4years | H | A | R |
| 2 | 7 | Female | 23 | RRMS | 1.5 | Interferon beta-1a (Avonex & Rebif) | 2months | 1year | L | I | R |
| 3 | 9 | Male | 32 | RRMS | 3 | Interferon beta-1a (Avonex & Rebif) | 2years | 5years | L | A | N |
| 4 | 11 | Female | 23 | RRMS | 2.5 | Interferon beta-1a (Avonex & Rebif) | 2years | 4years | L | I | R |
| 5 | 13 | Female | 45 | RRMS | 3 | Interferon beta-1a (Avonex & Rebif) | 1.5years | 15years | H | A | R |
| 6 | 15 | Male | 43 | RRMS | 2 | Interferon beta-1a (Avonex & Rebif) | 4years | 8years | L | I | R |
| 7 | 16 | Female | 25 | RRMS | 1 | Interferon beta-1a (Avonex & Rebif) | 3years | 9years | L | A | R |
| 8 | 29 | Male | 36 | RRMS | 3.5 | Interferon beta-1a (Avonex & Rebif) | 2years | 4years | H | A | R |
| 9 | 32 | Male | 32 | RRMS | 2.5 | Interferon beta-1a (Avonex & Rebif) | 1.5years | 5years | H | A | R |
| 10 | 35 | Female | 31 | RRMS | 3 | Interferon beta-1a (Avonex & Rebif) | 6months | 3years | H | A | N |
| 11 | 37 | Female | 29 | RRMS | 2.5 | Interferon beta-1a (Avonex & Rebif) | 6months | 1year | H | A | R |
| 12 | 38 | Female | 47 | RRMS | 3 | Interferon beta-1a (Avonex & Rebif) | 6months | 2years | H | A | N |
| 13 | 39 | Female | 37 | RRMS | 2.5 | Interferon beta-1a (Avonex & Rebif) | 2months | 1.5years | H | A | R |
| 14 | 42 | Female | 24 | RRMS | 2 | Interferon beta-1a (Avonex & Rebif) | 2years | 2years | H | A | R |
| 15 | 43 | Male | 20 | RRMS | 2.5 | Interferon beta-1a (Avonex & Rebif) | 1week | 2years | H | A | N |
| 16 | 50 | Female | 34 | RRMS | 3 | Interferon beta-1a (Avonex & Rebif) | 2months | 1year | M | A | R |
| 17 | 57 | Female | 31 | RRMS | 2.5 | Interferon beta-1a (Avonex & Rebif) | 2months | 8years | H | A | N |
| 18 | 59 | Male | 21 | RRMS | 4 | Interferon beta-1a (Avonex & Rebif) | 1week | 4years | M | A | R |
| 19 | 65 | Male | 23 | RRMS | 2 | Interferon beta-1a (Avonex & Rebif) | 2months | 2years | H | A | R |
| 20 | 66 | Female | 25 | RRMS | 1.5 | Interferon beta-1a (Avonex & Rebif) | 6months | 4years | H | A | R |
| 21 | 67 | Female | 42 | RRMS | 1.5 | Interferon beta-1a (Avonex & Rebif) | 1year | 7years | H | I | R |
| 22 | 69 | Female | 34 | RRMS | 2 | Interferon beta-1a (Avonex & Rebif) | 1month | 3years | H | I | R |
| 23 | 71 | Male | 21 | RRMS | 1.5 | Interferon beta-1a (Avonex & Rebif) | 2years | 6years | H | A | N |
| 24 | 78 | Male | 30 | RRMS | 1 | Interferon beta-1a (Avonex & Rebif) | 2years | 2years | L | I | R |
| 25 | 3 | Female | 33 | RRMS | 3 | Fingolimod (Gilenya) | 1year | 6years | M | A | N |
| 26 | 6 | Male | 47 | SPMS | 6 | Fingolimod (Gilenya) | 2weeks | 5years | H | A | N |
| 27 | 12 | Female | 46 | RRMS | 4.5 | Fingolimod (Gilenya) | 2years | 14years | H | A | R |
| 28 | 17 | Female | 42 | RRMS | 5.5 | Fingolimod (Gilenya) | 4months | 12years | H | A | N |
| 29 | 18 | Male | 23 | RRMS | 4.5 | Fingolimod (Gilenya) | 6months | 6years | H | A | R |
| 30 | 20 | Male | 32 | RRMS | 2.5 | Fingolimod (Gilenya) | 1.5years | 3years | L | A | R |
| 31 | 21 | Female | 30 | RRMS | 2 | Fingolimod (Gilenya) | 2months | 2years | H | A | N |
| 32 | 22 | Female | 36 | RRMS | 5.5 | Fingolimod (Gilenya) | 7months | 2.5years | H | A | R |
| 33 | 27 | Male | 37 | RRMS | 3 | Fingolimod (Gilenya) | 1year | 2years | H | I | R |
| 34 | 30 | Female | 37 | RRMS | 3.5 | Fingolimod (Gilenya) | 2years | 3years | H | A | R |
| 35 | 33 | Male | 23 | RRMS | 3.5 | Fingolimod (Gilenya) | 6months | 3years | H | A | R |
| 36 | 40 | Female | 43 | RRMS | 3 | Fingolimod (Gilenya) | 1year | 1.5years | H | A | R |
| 37 | 41 | Male | 23 | RRMS | 4.5 | Fingolimod (Gilenya) | 2months | 1year | H | A | N |
| 38 | 44 | Female | 22 | RRMS | 4 | Fingolimod (Gilenya) | 1year | 5years | M | I | R |
| 39 | 46 | Female | 32 | RRMS | 2 | Fingolimod (Gilenya) | 1year | 4years | H | A | R |
| 40 | 47 | Female | 39 | RRMS | 3 | Fingolimod (Gilenya) | 1year | 3years | H | A | R |
| 41 | 49 | Female | 28 | RRMS | 3.5 | Fingolimod (Gilenya) | 9months | 6months | H | A | R |
| 42 | 52 | Female | 37 | RRMS | 3.5 | Fingolimod (Gilenya) | 2weeks | 1.5years | H | A | N |
| 43 | 56 | Female | 41 | RRMS | 2.5 | Fingolimod (Gilenya) | 1year | 3years | H | I | R |
| 44 | 58 | Female | 38 | RRMS | 3.5 | Fingolimod (Gilenya) | 2months | 4years | M | A | R |
| 45 | 64 | Female | 21 | RRMS | 2 | Fingolimod (Gilenya) | 7months | 8years | H | I | R |
| 46 | 68 | Male | 38 | RRMS | 4 | Fingolimod (Gilenya) | 2months | 5years | H | A | N |
| 47 | 72 | Male | 42 | SPMS | 4 | Fingolimod (Gilenya) | 2months | 7years | L | A | R |
| 48 | 74 | Male | 26 | RRMS | 1.5 | Fingolimod (Gilenya) | 7years | 7years | M | I | R |
| 49 | 81 | Male | 25 | RRMS | 1 | Fingolimod (Gilenya) | 7months | 2years | H | A | R |
| 50 | 82 | Female | 29 | RRMS | 1.5 | Fingolimod (Gilenya) | 2years | 7years | H | I | R |
| 51 | 10 | Female | 35 | RRMS | 1 | Teriflunomide (Aubagio) | 4months | 6years | L | A | R |
| 52 | 14 | Female | 40 | RRMS | 2.5 | Teriflunomide (Aubagio) | 6months | 1year | L | A | R |
| 53 | 34 | Female | 45 | RRMS | 3 | Teriflunomide (Aubagio) | 1year | 8years | H | A | R |
| 54 | 45 | Female | 31 | RRMS | 2 | Teriflunomide (Aubagio) | 1year | 1.5years | L | I | R |
| 55 | 53 | Female | 21 | RRMS | 2.5 | Teriflunomide (Aubagio) | 2years | 2years | L | I | R |
| 56 | 2 | Male | 54 | RRMS | 6 | Interferon beta-1b(Betaferon) | 6months | 28years | H | A | N |
| 57 | 5 | Male | 49 | RRMS | 5 | Interferon beta-1b(Betaferon) | 7years | 7years | H | A | N |
| 58 | 8 | Female | 51 | RRMS | 3 | Interferon beta-1b(Betaferon) | 4years | 11years | L | I | R |
| 59 | 31 | Female | 22 | RRMS | 4 | Interferon beta-1b(Betaferon) | 3months | 5years | H | A | N |
| 60 | 36 | Female | 37 | RRMS | 2.5 | Interferon beta-1b(Betaferon) | 2weeks | 4years | L | A | N |
| 61 | 48 | Male | 34 | RRMS | 3 | Interferon beta-1b(Betaferon) | 2years | 7years | H | A | R |
| 62 | 63 | Female | 34 | RRMS | 1.5 | Interferon beta-1b(Betaferon) | 6years | 6years | L | I | R |
| 63 | 76 | Male | 28 | RRMS | 1 | Interferon beta-1b(Betaferon) | 2weeks | 8years | H | A | R |
| 64 | 80 | Female | 25 | RRMS | 1 | Interferon beta-1b(Betaferon) | 5years | 5years | L | I | R |
| 65 | 25 | Female | 36 | RRMS | 2 | Dimethyl Fumarate (Marovarex) | 8months | 2years | L | I | R |
| 66 | 26 | Male | 23 | RRMS | 3 | Dimethyl Fumarate (Marovarex) | 1year | 3years | H | A | R |
| 67 | 28 | Female | 28 | RRMS | 3.5 | Dimethyl Fumarate (Marovarex) | 1year | 2years | H | A | R |
| 68 | 60 | Female | 27 | RRMS | 4 | Dimethyl Fumarate (Marovarex) | 1year | 4years | H | A | R |
| 69 | 62 | Male | 42 | RRMS | 4 | Dimethyl Fumarate (Marovarex) | 2months | 5years | H | A | N |
| 70 | 70 | Male | 33 | RRMS | 1.5 | Dimethyl Fumarate (Marovarex) | 1year | 24years | H | I | R |
| 71 | 75 | Female | 25 | RRMS | 1.5 | Dimethyl Fumarate (Marovarex) | 4months | 1year | M | A | R |
| 72 | 77 | Male | 40 | RRMS | 4.5 | Dimethyl Fumarate (Marovarex) | 3years | 4years | H | A | N |

**Supplementary Table 2: Controls characteristics.**

| Control | Gender  Female = F  Male = M | Age |
| --- | --- | --- |
| 1 | F | 30 |
| 2 | F | 25 |
| 3 | F | 32 |
| 4 | F | 29 |
| 5 | M | 40 |
| 6 | M | 33 |
| 7 | F | 25 |
| 8 | F | 45 |
| 9 | M | 36 |
| 10 | F | 27 |

**Materials and methods**

## **Sample collection**

Blood samples were collected from 72 MS patients at the MS Unit, Nasser Institute for Research and Treatment following their written-informed consent and the ethical approval by the German University in Cairo Ethics Review Committee (Cairo, Egypt). The study protocol was approved by the Ethics Review Committee of the German University in Cairo (Approval Code: PTX-2020-06, Approval Date: 28/6/2020) and was conducted in accordance with the principles of the 1975 Declaration of Helsinki. The inclusion criteria included patients diagnosed with any MS type, aged between 20-60 years, and undergoing active treatment. Exclusion criteria were patients experiencing an active relapse during the sampling period. 10 age matched healthy controls were also included.

## **Ficoll density gradient technique**

10 ml blood was collected in EDTA tubes and 3 ml plasma was stored for further analysis. Within 4 hours of sampling, peripheral blood mononuclear cells (PBMCs) were isolated from whole blood using Ficoll (cat. no.10771; Sigma-Aldrich, USA) according to manufacturer’s guidelines. Blood was diluted 1:1 with the wash mix containing Rosewell Park Memorial Institute Medium-1640 (RPMI 1640, cat. no. SR263-10L; Serox, Germany), supplemented with L-glutamine, Phenol red, 5% FBS (cat. no. 10270098) and 1% Penicillin/Streptomycin (cat. no. 15140122; both from Applied Biosystems; Thermo Fisher Scientific, Inc.). Diluted blood was carefully layered on top of 3 ml Ficoll and centrifuged at 4000 RPM for 30 minutes. The collected cells from the buffy layer underwent two washes and viable cell count was determined. Cells were cryopreserved at -80˚C with a concentration of 10^7^ cells/ml in a mixture of 50% v/v supplemented media, 40% v/v FBS and 10% v/v dimethyl sulfoxide (DMSO; cat. no. D12345; Applied Biosystems; Thermo Fisher Scientific Inc.) for future analysis. Samples were stored for up to 6 months and upon thawing, viability was assessed using 0.4% Trypan blue (cat. no. 15250061; Thermo Fisher Scientific, Inc.) with an acceptable viability of >80%.

## **Isolation of CD14+ monocytes by negative depletion using magnetic nanobeads**

Frozen PBMCs were thawed at 37˚C and transferred to 10 ml supplemented media then centrifuged at 300 xg for 5 min at room temperature. The isolation of CD14+CD16- monocytes from PBMCs was carried out through negative depletion using the MojoSort™ Human CD14+ Monocytes Isolation Kit (cat. no. 480047), MojoSort™ Buffer (5X) (cat. no. 480017) and MojoSort™ Magnet (cat. no. 480019; all Biolegend, Inc), following the manufacturer’s instructions. The resultant pure CD14+ monocytes were subsequently centrifuged (300 xg for 5 min at room temperature) and re-suspended in culture media.

1. **Cell culture and differentiation**

Freshly isolated CD14+ monocytes were resuspended at 1x10^6^ cells/ml in M2 macrophage differentiation medium consisting of supplemented media containing macrophage colony stimulating factor (M-CSF:100 ng/ml; cat. no. 100-03, Schenendoah Biotechnology, US) and Interleukin 4 (IL-4:10 ng/ml; cat. no. 100-09, Schenendoah Biotechnology, US). The cells were cultured on a 48 well plate (10,000 cells/well) and were incubated at 37˚C and 5% CO_2._ Media were refreshed every other day and by day 4, Interleukin 10 (IL-10 :10 ng/ml; cat. no.100-83, Schenendoah, US) was added to the culture. On day 7, the cells were harvested and ready to be transfected.

1. **Flow cytometry**

Confirmation of CD14+ monocytes isolation and M2 cells differentiation was carried out through flow cytometry on the collected population. Cells were first dissociated, followed by preparation of single-cell suspension (240,000 cells/tube). Then washed with 2 ml (PBS 1% FBS) and centrifuged at 350 xg for 5 min and the supernatant was discarded. Finally, the cells were incubated with 1.2 μg anti-CD163 FITC (cat. no: sc-33715; Santa Cruz Biotechnology) for 30 min at 4˚C followed by a washing step and acquisition. Flow cytometry analysis was conducted using CytoFLEX benchtop flow cytometer (Beckman Coulter Inc.) gating for the CD-163+ population. The acquired fluorescence data were then analyzed using the CytExpert software (version 2.3.3.84; Beckman Coulter Inc.) to assess the sample’s purity. With regard to the isolation process and the size scatter of the resultant populations, of note, a small percentage of cells (30%), were remaining monocytes and other T-cells that were not completely depleted.

1. **Transfection**

Differentiated M2 macrophages harvested on day 7 were treated with 2 μl siRNA of lncRNA AFAP1-AS1 (Hs_MGC1098_3 FlexiTube siRNA; cat. no. SI04767511, Qiagen GmbH) diluted in 60 μl serum-free culture medium. In addition, scrambled small interfering RNAs (Scr-siRNA) were used as negative controls to account for potential non-specific effects of siRNA transfection. (Scr-siRNAs) (cat. no. 1027292, Qiagen GmbH). The appropriate amount of HiPerfect Transfection Reagent (cat. no. 301704; Qiagen GmbH) was added (1 μl) to the diluted siRNA then mixed by vortexing. The siRNA-HPTR mixture was incubated for 5–10 min at room temperature allowing the formation of transfection complexes. The complex was added dropwise onto the cells and the plates were swirled gently to ensure uniform distribution of transfection complexes. All transfection experiments were conducted in triplicates as per the manufacturer’s instructions, with each experiment repeated three times. Cells treated with transfection reagent only were designated as mock cells, cells transfected with Scr-siRNAs (negative controls) were designated as siNC and cells transfected with siRNA against AFAP1-AS1 were designated as siAFAP1-AS1 cells. To ensure efficient transfection, cells were incubated for 6 hours then, 140 μl complete culture media of RPMI containing serum and antibiotics were added to the cells and incubated under normal conditions (37˚C with 5% CO_2_). Cells were lysed 48 h post transfection for RNA extraction and subsequent analysis of gene expression.

1. **RNA isolation**

RNA was extracted using the RNeasy Minikit (cat. no. 74104; Qiagen GmBH) following the recommended extraction protocol. Up to 1 x 10^7^ cells, are disrupted in 350 µl buffer RLT and homogenized and mixed by vortexing or pipetting. 1 volume of prepared 70% ethanol was added to the homogenized lysate and mixed well by pipetting. Up to 700 μl of the sample was transferred to the RNeasy spin column placed in a 2 ml collection tube and washed twice with 700 µl RW1 and 500 µl RPE respectively and centrifuged, with the flow discarded each time. The column was then placed in a 1.5 ml collection tube and 40 µl RNase-free water was added directly to the spin column and centrifuged for 1 min at 12000 RPM to elute RNA. Total RNA was stored in -80˚C for later quantification of genes by PCR. Assessment of RNA concentration and purity was carried out using Nanodrop, ensuring an A_260/280_ ratio falling within the acceptable range of 1.9-2.2. For each sample, a total RNA ranging from 30-50 ng was utilized. RNA integrity was tested by 18s rRNA band detection using 1% agarose gel electrophoresis.

1. **Reverse transcription of total mRNA into cDNA**

The total extracted RNA was reverse-transcribed into single-stranded cDNA using the high-capacity cDNA reverse transcription kit (cat. no. 4368814; Applied Biosystems; Thermo Fisher Scientific, Inc.) as per the manufacturer’s instructions. Each component of the kit and the extracted RNA were thawed on ice and mixed by vortexing to ensure appropriate resuspension. The reaction mix was prepared by adding 3.7 μl nuclease free water, 3 μl RT buffer, 1 μl RT Random primers, 1 μl RNase inhibitor, 0.8 μl dNTP mix and 0.5 μl MultiScribe Reverse Transcriptase. Total tube volume of each reaction was 20 μl with 1:1 ratio (reaction mix: total RNA). Finally, all reaction tubes were placed in a thermo cycler with a heated lid whose thermal profile was adjusted as per the manufacturer’s protocol. All cDNA samples were stored in the -20˚C freezer until qRT-PCR analysis was performed.

1. **Quantitative polymerase chain reaction (RT-qPCR) of AFAP1-AS1**

Subsequently, the relative expression of AFAP1-AS1 normalized to GAPDH as a housekeeping gene, was quantified and amplified through TaqMan RT-quantitative polymerase chain reaction (qPCR; Assay IDs: 4331182, 4326317E, Applied Biosystems; Thermo Fisher Scientific, Inc.) for AFAP1-AS1 and GAPDH respectively using a StepOne™ Real-Time PCR instrument (Applied Biosystems; Thermo Fisher Scientific, Inc.). Probes used for AFAP1-AS1 were labeled with FAM reporter dye while the GAPDH was labeled with the VIC reporter dye. For each sample, a reaction mix consisting of 4 μl nuclease-free water, 10 μl Premix Ex Taq^TM^ (Probe qPCR), 1 μl TaqMan target gene expression assay (x20) and 1 μl GAPDH (VIC) was prepared according to the manufacturer’s instructions, and 4 μl of the respective cDNA was added. The RT-qPCR run was conducted in the standard mode and consisted of two stages: an initial 10-minute stage at 95˚C for the activation of the Taq-polymerase enzyme, followed by a second stage of 40 amplification cycles (15 seconds at 95˚C and 60 seconds at 60˚C). All PCR reactions, including controls, were carried out in triplicates and the relative expression of genes was determined using the 2^-ΔΔCq^ method.

1. **Protein Quantification of MMP9, CCL5 and CXCL10 in serum samples using ELISA**

Protein release of MMP9, CCL5 and CXCL10 in cell culture supernatants was conducted following the manufacturer’s instructions using the human MMP9 ELISA kit (cat. No. MBS175780) the human CCL5 ELISA kit (cat. No. MBS824711) and the human CXCL10 ELISA kit (cat. No. MBS8123888; all from MyBioSource, Inc). The test principle applied in this kit is Sandwich enzyme immunoassay. The microtiter plate provided has been pre-coated with an antibody specific to MMP9, CCL5, or CXCL10. In brief, 100 μl of each serially diluted protein standard, samples or blank was added into the appropriate wells according to the manufacturer’s protocol and incubated for 2 hours then the wells were washed with 250 μl of 1× Wash Solution per well. After washing, 100 μl of Biotinylated Antibody working solution was added to each well and incubated for 1 hour at 37˚C. The wells were washed again and 100 μl of Streptavidin HRP working solution was added to each well and incubated for 1 hour at 37˚C. After washing, 90 μl of TMB Substrate Solution was added to each well and incubated for 15-30 minutes at 37˚C according to the exact protocol of each protein and protected from light. Color development on the plate is monitored for 30 min at room temperature, then the stop solution was added to terminate the reaction, and the absorbance was analyzed for each microwell for both standards and samples at 450 nm wavelength. The results are calculated by constructing a standard curve plotting the mean OD and concentration for each standard.

1. **Statistical analysis**

Gene expression results were represented in relative quantitation (RQ). All experiments were carried out in triplicates and data was presented as mean ± standard error of mean (SEM). Prior to statistical analysis, data distribution was assessed for normality using the Shapiro–Wilk test, and homogeneity of variance was evaluated using Levene’s test. Since the data satisfied the assumptions of normality and equal variance, Student's t-test and One-way ANOVA were applied without data transformation. Given the small sample sizes per group (n=3–4), the results of normality testing should be interpreted with caution. However, parametric tests were deemed appropriate based on the available data and consistent with similar studies in the field. Statistical analyses were performed using GraphPad Prism 7.04 software (GraphPad Software, Inc.). For comparison between two different studied groups, Student's unpaired T-test was employed. One Way Anova followed by Dunette’s test of multiple comparison was used for the comparison between more than two different studied groups. A significance level of P<0.05 was considered indicative of a statistically significant difference (**** = P<0.0001, ***=P<0.001, **=P<0.01, *=P<0.05 and ns=statistically not significant).
